# Measurement-based care for suicidal youth: Outcomes and recommendations from the Services for Teens At Risk (STAR) Center

**DOI:** 10.1371/journal.pone.0284073

**Published:** 2023-04-06

**Authors:** Sarah E. Victor, Rachel H. Salk, Giovanna Porta, Edward Hamilton, Kelsey Bero, Kim Poling, David A. Brent, Tina R. Goldstein

**Affiliations:** 1 Department of Psychological Sciences, Texas Tech University, Lubbock, Texas, United States of America; 2 Department of Psychiatry, University of Pittsburgh School of Medicine, Pittsburgh, Pennsylvania, United States of America; 3 Western Psychiatric Hospital, University of Pittsburgh Medical Center, Pittsburgh, Pennsylvania, United States of America; Johns Hopkins School of Medicine: Johns Hopkins University School of Medicine, UNITED STATES

## Abstract

Measurement-based care has demonstrable benefits, but significant implementation barriers slow dissemination in real-world clinical settings, especially youth behavioral health care. Here, we describe use of measurement-based care in a specialty clinic offering a continuum of outpatient care for suicidal youth. We characterize strategies used to facilitate measurement-based care in this population and ways in which challenges to implementation have been addressed. We examined adherence to measurement-based care procedures relative to treatment engagement data from electronic medical records, as well as data from clinicians regarding acceptability and utility of measurement-based care. Results suggest that measurement-based care is both feasible and acceptable for use with suicidal youth. Here we provide future directions in measurement-based care in this, and other, behavioral health settings.

## Introduction

Measurement-based care (MBC) involves “the systematic use of symptom rating scales to drive clinical decision-making” [1] and is required for Joint Commission accreditation in behavioral healthcare [2]. MBC is associated with improved patient outcomes in mental healthcare settings [3], including improved response and remission rates in the treatment of adult depression [4, 5]. Similar, although sparser, data support improved patient outcomes when MBC is used with youth [6]. MBC improves identification of stalled progress or clinical deterioration among patients, facilitating needed changes in treatment to improve patient outcomes [7], and enhances patients’ engagement with treatment [8]. Further, MBC offers important feedback to clinicians about their own practices and demonstrates the efficacy of behavioral health services to third-party payers, such as health insurance companies [9].

MBC has been used in a variety of research and clinical settings to examine treatment efficacy and outcomes for depression among adults [4, 5, 10] and youth [11–13]. Relatively less work has examined MBC in the clinical management of suicidal thoughts and behaviors [14]. This gap is concerning, given the ethical and practical necessity of regular monitoring of suicide risk in clinical practice [15]. Suicidal thoughts and behaviors vary markedly over time among high-risk individuals [16], which necessitates frequent assessment to inform decisions about imminent risk management in the case of suicidal crises [17]. Thus, MBC with suicidal youth informs critical clinical decisions, such as communication with caregivers regarding safety and the potential need for psychiatric hospitalization [18]. The most well-known example of MBC in the treatment of suicidal patients is the use of “diary cards” in dialectical behavior therapy (DBT). DBT is a well-validated treatment for suicide and self-harm across diagnostic groups [19, 20]. Diary cards help monitor patient progress and inform treatment planning; data drawn from diary cards has high utility even outside of adherent DBT provision [21].

Although the literature supporting MBC in behavioral health broadly is substantial, there remain significant gaps in its study and implementation. First, much of the support for MBC is from adult treatment settings, such as Veterans Affairs centers [22] and academic medical centers participating in nationally organized consortia [14], or from adult depression research trials [4, 5]. Even in these contexts, research on MBC for suicide-related phenomena has been rare, and typically involves very infrequent administration of suicide assessments relative to what would be typical in MBC [14]. Research that has described MBC in adolescent mental health contexts has been primarily drawn from depression treatment trials, such as TORDIA [23, 24] among others [11, 13]. In some cases, work has been published describing platforms, tools, and/or strategies that could be used to facilitate youth MBC in naturalistic treatment settings [25–28], but without empirical data to characterize its implementation in specific treatment environments. Some limited data have been presented to support MBC with youth in a school-based mental health system [29], a partial hospitalization program for mood and anxiety disorders [30], and a first-episode psychosis program [31]; however, to our knowledge, no research has focused on MBC with suicidal youth in particular.

Thus, this paper characterizes the use of MBC at the Services for Teens At Risk (STAR) Center, a well-established program providing outpatient and intensive outpatient (IOP) mental health treatment to suicidal youth. MBC is a core component of regular clinical services at STAR-Center that informs individual patients’ care and overall program development. First, we provide an overview of STAR-Center’s patient population, treatment approach, and use of MBC, as well as how challenges to MBC implementation have been addressed. Second, we summarize clinician-report data on the feasibility and utility of MBC at STAR-Center, along with evidence of successful implementation of MBC based on de-identified patient data. Finally, we describe lessons learned through STAR-Center’s use of MBC and future directions of MBC with this population, with the goal of facilitating dissemination and implementation of MBC in diverse mental health treatment settings that provide care for suicidal youth.

## What is STAR-Center?

STAR-Center was founded in 1986 as a suicide prevention program for youth [32]. Supported by the Commonwealth of Pennsylvania, Western Psychiatric Hospital, and the Department of Psychiatry at the University of Pittsburgh, STAR-Center provides assessment and intervention services to suicidal youth and young adults, as well as community outreach and suicide postvention services [32]. As the STAR-Center only expanded services to young adults in 2017, and MBC data were not available for analysis during the drafting of this manuscript, the remainder of the manuscript will focus specifically on describing services provided to youth through age 18.

STAR-Center patients complete an extensive intake assessment to determine appropriateness for STAR-Center services, which includes thorough suicide risk assessment and psychiatric diagnosis. Youth appropriate for STAR-Center services are then assigned to treatment along a continuum of care, beginning either traditional outpatient psychotherapy services (one hour/week) or IOP treatment (nine hours/week) depending on clinical severity, need, and patient preference. IOP treatment includes skills group and individual psychotherapy with master’s level clinicians and medication management with a psychiatrist and psychiatric nurse. Family sessions are conducted as clinically appropriate. Patients can move between levels of care, for instance, beginning with IOP services and stepping down to traditional outpatient psychotherapy when symptoms are less acute. STAR-Center also offers a monthly continuation/maintenance group for patients who no longer require weekly support. In some cases, patients complete the IOP portion of treatment and continue ongoing treatment with community providers outside of STAR-Center. Treatment at STAR-Center uses evidence-based practices, including DBT and cognitive behavior therapy modalities, informed by each patient’s case conceptualization [33].

Beyond clinical care, STAR-Center has a strong clinical-research infrastructure, which facilitates scientific research to improve our knowledge and treatment of suicidal youth. Further, STAR-Center provides extensive training support to mental health professionals and students seeking to develop competency and expertise in suicide risk assessment and management, including masters’ level clinician trainees, predoctoral clinical psychology interns, postdoctoral fellows, and medical students, interns, and residents.

## Measurement-based care at STAR-Center

As part of standard clinical care, patients, their caregivers, and clinicians complete numerous assessments over the course of treatment (see Table 1 [34–53]). Patients and caregivers complete an extensive intake assessment, including clinician-administered interviews and validated self-report and informant-report questionnaires. The clinician-administered intake evaluation includes the Suicide Circumstances Schedule [54], sections of the Columbia Suicide Severity Rating Scale [55], the Kiddie Schedule for Affective Disorders and Schizophrenia Depression Rating Scale [56], the Clinical Global Impressions Scale [57], and the Child Global Assessment of Functioning [58]. A shorter battery of self-report (patient-rated) measures is administered at regular intervals during treatment (see below for specific intervals) using a HIPAA-compliant secure web-based survey system accessed on tablets to facilitate ease of data collection and scoring. Caregiver-rated (informant-report) measures are limited to the intake assessment battery given that measures are completed at the time of the session, and in our population, caregivers infrequently attend visits after the initial intake assessment. Patients enrolled in the IOP program complete MBC assessments weekly, as well as IOP diary cards for each day they attend the program; patients seen weekly or bi-weekly in the traditional outpatient program complete assessments monthly. The high acuity and symptom severity among youth in IOP, taken with the IOP treatment intensity, render week to week changes in MBC data relevant to reflect improvement or clinical worsening. These data are regularly reviewed to determine acute treatment response and related determinations regarding appropriate level of care and risk management. These data may also be used in the process of insurance authorizations for IOP level of care to demonstrate clinical need. For youth in outpatient (weekly to biweekly) level of care, monthly MBC data enable us to note trends and treatment response while also minimizing respondent burden. Clinicians treating outpatients have the capacity to initiate interim assessment as clinically indicated. Continuation/maintenance group is held on a monthly basis, and assessments are completed at each group session (i.e., monthly). Clinicians also complete rating scales assessing depression symptoms, clinical severity, clinical improvement, suicidality and self-injury, and medication changes throughout treatment; clinician ratings are generated regardless of patient self-report completion.

**Table 1:** Selected STAR-Center MBC assessment tools and their psychometric properties.

Measure	Construct	# Items	Scoring	Factors	Psychometric Support
SMFQ	Depression	13	Item: 0–2	1	[34–36]
			Total: 0–26		
			Clinical: 12–26		
SCARED	Anxiety	41^a^	Item: 0–2	5^b^	[37–39]
			Total: 0–82		
			Clinical: 25–82		
CALS	Affective Lability / Emotion Dysregulation	20	Item: 0–4	2^c^	[40–42]
			Total: 0–80		
			Clinical: 10–80		
PSQI	Sleep	10	Item: 0–3	1	[43–45]
			Total: 0–21		
			Clinical: 6–21		
CRAFFT	Substance Use	9	Item: 0–1	1	[46–48]
			Total: 0–9		
			Clinical: 2–9		
ARI	Irritability	7	Item: 0–2	1	[49–51]
			Total: 0–14		
			Clinical: 3–14		
ASQ^d^	Suicidality and Self-Harm	5	Item: 0–1	NA	[52–53]
			Total: NA		
			Clinical: NA		

SMFQ = Short Mood and Feelings Questionnaire; SCARED = Screen for Child Anxiety Related Disorders; CALS = Children’s Affective Lability Scale; PSQI = Pittsburgh Sleep Quality Index; CRAFFT = Car, Relax, Alone, Forget Friends, Trouble; ARI = Affective Reactivity Index; ASQ = Ask Suicide Screening Questions.

^a^ For the SCARED, the full (41-item) version is given at baseline and for MBC (repeated use) for patients with a primary anxiety disorder diagnosis; all other patients complete the 5-item short form for MBC (repeated use).

^b^ The 41-item version of the SCARED yields five factors: panic/somatic symptoms, general anxiety, separation anxiety, social phobia, and school phobia.

^c^ The CALS yields two subscales which assess behaviors related to irritability/depression and disinhibited behaviors.

^d^ ASQ questions have been modified to assess death ideation, suicide ideation, suicide planning, suicide attempts, and non-suicidal self-injury, and are used (along with IOP diary cards) to identify high-risk suicide responses requiring clinician “flags” for patients.

The entirety of STAR-Center’s treatment team (see below) meets weekly to review MBC data. Upon completion, patient MBC data are securely transmitted to an encrypted database, where assessments are scored and compiled with previously collected data. To facilitate comparability across measures with different scoring systems, results are scaled to a 0–100 interval based on percentage of the measure’s maximum score. Results for all constructs are then displayed using line graphs to examine changes in each symptom domain over time. Data are then used to examine patient progress, deterioration, and/or stagnation, and MBC-based treatment changes are made as needed (e.g., increased/decreased level of care, increased/decreased medication dosage, trial of alternative medication(s), transition to a different therapist or program). Data are also reviewed with the patient (and caregivers, as appropriate) on a regular basis in psychotherapy and medication management appointments.

**Fig 1:**
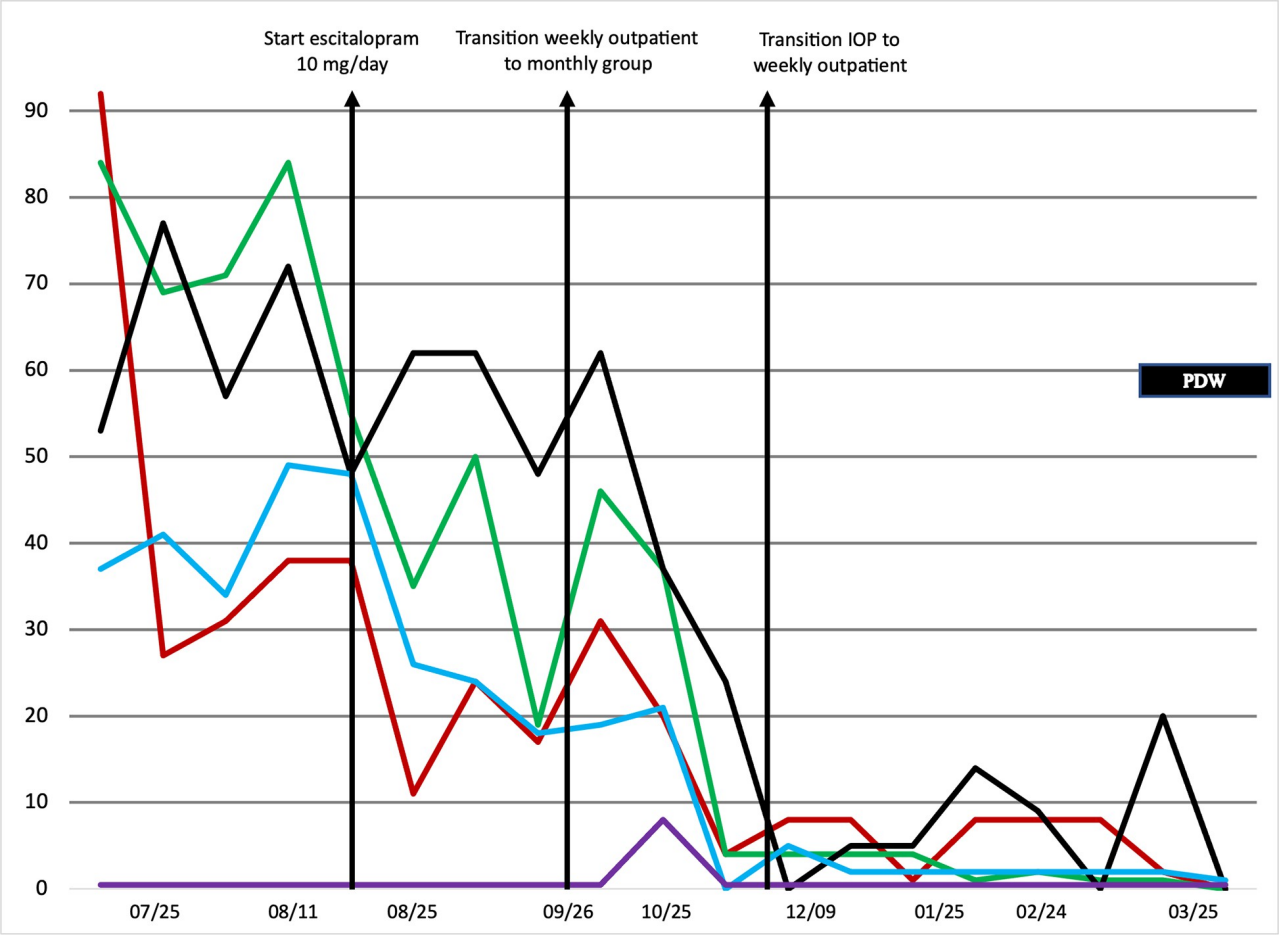
De-identified example of MBC data display for treatment team meeting. Scores are scaled as a percentage of the maximum score for each measure (see Table 1). Lines indicate scores on the following scales: Mood: red (Short Mood and Feelings Questionnaire); Anxiety: green (Screen for Child Anxiety Related Disorders); Sleep: black (Pittsburgh Sleep Quality Index); Mood Lability: blue (Children’s Affective Lability Scale); Substance Use: purple (Car, Relax, Alone, Forget Friends, Trouble). The black box labeled “PDW” is an example of a “flag” based on responses to the ASQ at the most recent assessment, in this case indicating endorsement of passive death wishes.

Fig 1 shows a de-identified, annotated example of how data are displayed for treatment-team meetings and patient review. At the start of IOP, this patient reported very high levels of depression and anxiety, moderate sleep problems and affective lability, and minimal difficulties with substance use. During IOP treatment, their depression decreased significantly, but their anxiety, sleep problems, and affective lability remained relatively stable. The patient was prescribed 10 mg/day of escitalopram (first vertical arrow), at which point their anxiety and affective lability decreased. The patient’s depression symptoms remained low for several weeks, leading to discharge from IOP to weekly outpatient psychotherapy and medication management (second vertical arrow). The patient continued to improve, including resolution of their sleep difficulties, at which point they were discharged from weekly individual care to monthly continuation/maintenance group treatment (third vertical arrow). The patient remained well over the following several months of continuation/maintenance group.

## Addressing challenges in measurement-based care

Several important challenges have been identified in the implementation of MBC, which have been addressed in a variety of ways at STAR-Center. MBC assessments, like all measures used in health care settings, must be both reliable and valid for their intended purpose and developmentally appropriate [59]. To decrease patient burden, MBC assessments must also be brief and easy to complete [60]. Finally, MBC assessments must be sensitive to change over time [61].

Thus, STAR-Center uses brief versions of well-validated measures appropriate for youth MBC. Each measure administered repeatedly ranges from 5 to 20 items, for a total completion time of 10–20 minutes per follow-up assessment; the intake battery takes approximately 25 minutes. Further, each measure has been validated with youth, and exhibits sensitivity to change over treatment (see Table 1).

Measures have been digitized for tablet-based access; patients typically complete measures via web-link in the waiting room prior to their appointment. Of note, our health system quickly transitioned to provision of service via telehealth in response to local, state, and national mandates during the COVID-19 pandemic. For all telehealth visits, the treating clinician sent a web-link via email or text message (based on family preference) separately to the youth (for intake and ongoing monitoring) and caregiver (for intake only) at the time of the scheduled session. The clinician would re-send the link if the assessment battery was not completed within 5 days of the completed session. Trends in MBC assessments occurring since the onset of the COVID-19 pandemic will be discussed in a separate manuscript.

Second, any MBC strategy requires significant buy-in from clinical staff and patients. Clinician burden and lack of investment is a significant obstacle to MBC in routine clinical care [62], and lack of patient buy-in contributes to missed assessments and limited utility of completed assessments in improving patient outcomes [63]. At STAR-Center, clinical staff have been involved throughout the process of MBC-related decision-making, for instance, in the choice of measures and the format for display of data. Clinicians complete brief surveys and participate in open discussions to gauge the utility of MBC assessments; two STAR-Center clinicians participate in twice monthly meetings to incorporate staff feedback into MBC-related changes. Second, staff clinicians receive ongoing education and training related to MBC during weekly meetings and MBC-specific trainings. Finally, senior clinical staff model the use of MBC data to make treatment decisions, as well as how to review and discuss MBC data with patients, during weekly treatment-team meetings. Similar strategies have been used at STAR-Center to enhance patient engagement. Patients are educated about MBC and its role in their treatment during their orientation to services at STAR-Center and emphasized repeatedly during treatment. Clinicians set a positive tone regarding MBC, for instance, by showing investment in patients’ understanding and completion of MBC measures throughout their treatment. Finally, when reviewing data with the patient, clinicians highlight connections between changes in treatment and changes in symptoms, demonstrating the value of MBC.

Third, timely, regular feedback given to both clinicians and patients is critical to MBC [64]. At STAR-Center, MBC is integrated into all documentation, case discussions, and case conceptualizations; MBC intake data are included in all patients’ initial assessment summaries, and clinicians are encouraged to include patient MBC assessment scores in weekly notes. Digitized MBC assessments ensure results are scored and analyzed in real-time, facilitating prompt review and response to patient-reported symptoms and/or suicide risk. Clinicians can view patients’ scores on self-report measures prior to meeting with the patient using a clinician dashboard, allowing them to use MBC data to structure subsequent patient encounters. Further, clinical staff are encouraged to share MBC results with patients as clinically appropriate; once patients are accustomed to the MBC system, they often ask clinicians to view their results during meetings. Patients are regularly shown their recent and historical data using line graphs, to demonstrate symptom changes over time in an easy-to-digest format.

Finally, managing suicide risk assessment and planning is a critical component of all psychiatric care, but is especially important at STAR-Center, where patients are specifically referred due to acute suicidality. Whenever a patient completes MBC assessments, the treating clinician is required to check responses regarding suicidality immediately, while the patient is still physically present at the clinic. Patients enrolled in IOP who report suicidal planning, a suicide attempt, or non-suicidal self-injury on their daily diary cards and/or weekly MBC assessment are “flagged” for further assessment by the lead IOP clinician; regardless of treatment modality, an email alert is sent automatically to the patient’s primary clinician and the clinical supervisor that details specific high-risk response(s).

## Evidence of MBC feasibility and utility at STAR-Center

Two sets of data were analyzed to examine the implementation of MBC at STAR-Center. Data were entered and examined using SPSS version 25. First, clinical staff at STAR-Center (*N* = 17) completed anonymous surveys to gather information about perceived benefits and costs of MBC in routine clinical practice using a measure adapted from a similar survey assessing perceptions of MBC in physical therapy [65]. Participants were given a number of statements describing possible benefits to, or problems with, MBC, and were asked to respond as to whether they definitely agreed, agreed somewhat, or disagreed. The survey was approved for distribution through the University of Pittsburgh’s Quality Improvement office, and determined not to require Institutional Review Board approval.

Clinical staff at STAR-Center were primarily cisgender women (*n* = 13, 76.47%) between the ages of 25 and 49 (*n* = 12, 70.59%). All staff held masters (*n* = 11, 64.71%) or doctoral (*n* = 6, 35.29%) terminal degrees, and fields of study included psychiatry (*n* = 5, 29.41%), counseling (*n* = 4, 23.53%), psychology (*n* = 3, 17.65%), social work (*n* = 3, 17.65%), and nursing (*n* = 2, 11.76%). Of the clinicians who worked (or planned to work in the future) outside of STAR-Center (*n* = 14), a majority (*n* = 9, 64.29%) rated themselves as very likely or extremely likely to use MBC in another clinical setting. Clinicians were asked about the number of years using MBC, at STAR-Center or elsewhere; data were binned to ensure anonymity given potential for reidentification. Most clinicians indicating using MBC for 1 to 4 years (*n* = 6, 40%) or 5 to 10 years (*n* = 4, 26.67%), with fewer indicating less than 1 year (*n* = 1, 6.67%), 11 to 30 years (*n* = 2, 13.33%), or a qualitative description such as “long” (*n* = 2, 13.33%).

Clinicians were asked about potential benefits and problems related to the use of MBC at STAR-Center (see Table 2). All surveyed definitely or somewhat agreed that MBC helped to direct patients’ care, attain better patient outcomes, and help motivate and encourage patients; clinicians were divided with respect to whether MBC decreased rates of denial for care from third-party payers. The most commonly endorsed problems with MBC were that they take too much time for patients to complete, can be difficult for clinicians to interpret, and confusing to patients. Importantly, these perceptions are inclusive of all components of MBC used at STAR-Center, such as paper diary cards, even though these measures are not integrated into the Research Registry system used to compute estimates of MBC completion rates.

**Table 2:** STAR-Center clinician ratings of MBC benefits and challenges (n = 17 clinicians).

	Definitely Agree *n* (%)	Agree Somewhat *n* (%)	Disagree *n* (%)
MBC Benefits:			
Attaining better patient outcomes	14 (87.5)	2 (12.5)	0 (0.0)
Helping to direct the plan of care	13 (76.47)	4 (23.53)	0 (0.0)
Enhancing communication between therapist & patient	12 (70.59)	4 (23.53)	1 (5.88)
Helping to focus choice of interventions	11 (64.71)	5 (29.41)	1 (5.88)
Helping to motivate and encourage patients	10 (58.82)	7 (41.18)	0 (0.0)
Enhancing communication with third-party payers, physicians, and other providers	6 (35.29)	8 (47.06)	3 (17.65)
Decreasing the rates of denial from third-party payers	5 (29.41)	5 (29.41)	7 (41.18)
MBC Problems:			
They are difficult to interpret (e.g. do not know what norms are, how scores relate to severity, or what a clinically important change may be)	3 (17.65)	9 (52.94)	5 (29.41)
They take too much time for patients to complete	2 (11.76)	10 (58.82)	5 (29.41)
They take too much of clinicians’ time to analyze/calculate/score	2 (11.76)	3 (17.65)	12 (70.59)
They are confusing to patients	1 (5.88)	10 (58.82)	6 (35.29)
They are difficult for patients to complete independently	1 (5.88)	4 (23.53)	12 (70.59)
They are not sensitive to the cultural/ethnic concerns of many patients	0 (0.0)	6 (37.5)	10 (62.5)
They make patients anxious	0 (0.0)	5 (29.41)	12 (70.59)
They require too high a reading level for many patients	0 (0.0)	4 (25)	12 (75)
They require more effort than they are worth	0 (0.0)	3 (17.65)	14 (82.35)
They do not contain information that helps to direct the plan of care	0 (0.0)	2 (11.76)	15 (88.24)
They provide information that is too subjective to be useful	0 (0.0)	1 (5.88)	16 (94.12)

Scale items adapted from a measure of MBC in the context of physical therapy [65].

Additionally, we compared a representative sample of MBC data approved for research analysis with data from electronic medical records (EMR) to determine how regularly MBC assessments were gathered during routine clinical care, relative to MBC assessments expected based on actual clinic attendance. Patients at STAR-Center have the option to permit their MBC data to be used for research purposes; those who consent have their de-identified MBC data entered into a Research Registry. The University of Pittsburgh Institutional Review Board approved the study (IRB#9912102). Written consent was obtained from participants in the STAR-Center Research Registry. EMR data were extracted and de-identified by STAR-Center staff with appropriate clearance to access EMR, to facilitate cross-comparison with MBC data without compromising patient confidentiality. MBC and EMR data were examined for a seven-month period (1/1/2018-6/31/2018). As patients may have started treatment prior to, or continued treatment after, the selected period, data presented below likely underestimate the total MBC data collected on these patients. Rates of missing data from EMR are unavailable due to the nature of the dataset being examined. Raw data were not transformed on the basis of outliers or any other method. Sample size was determined by available data over the examined period and not based on a priori power analyses.

Given variations on the measures administered to specific patients (see Table 1), as well as the addition of some measures over time, MBC assessments were counted as present if at least one self-report measure was completed within the expected MBC battery for that time window. During the selected interval, 374 patients were seen at STAR-Center, and 340 (90.91%) consented to join the Research Registry. Of those 340 patients, 326 (95.88%) had at least one MBC assessment, and 230 (67.65%) had at least 2 (*M* = 3.54, *SD* = 2.59). Based on EMR data, the average number of completed assessments per patient was 3.54 (*SD* = 2.59), which is slightly lower than the expected number of assessments per patient (*M* = 3.70, *SD* = 2.54). Importantly, the number of expected assessments will not match the number of patient visits attended, as MBC measures are not administered at every visit, depending upon the patient’s level of care (see above for expected assessment intervals). Overall, across all patients, 84.10% (*n* = 1153/1371) of expected assessments were collected. A total of 227 patients (66.67%) had at least the expected number of MBC assessments; it was possible for patients to have more than the expected number of MBC data points, if they completed MBC measures more frequently than required (for instance, completing MBC measures twice in one week of IOP treatment, even though only one administration per week is expected). Of those who were missing assessments (*n* = 113, 33.24%), most were missing only one (*n* = 64, 56.64%), with a small proportion missing two (*n* = 30, 26.55%) or three (*n* = 19, 16.81%) MBC assessments.

Rates of adherence to MBC data collection procedures varied by treatment modality. High rates of completion were found for intake appointments (93.81%, *n* = 197/210), IOP appointments (96.79%, *n* = 452/467), and monthly outpatient continuation/maintenance group (93.18%, *n* = 82/88). The completion rate was significantly lower for patients seen on an outpatient basis weekly (69.64%, *n* = 422/606).

Because data were collated on the basis of attended treatment sessions listed in the EMR system (i.e., data were not counted as missing if the patient did not receive services), missed assessments were due either to the clinician failing to administer the measures, or the patient refusing to complete them. Although this information is not available in this dataset, clinical experience in this setting suggests that patient refusal is quite rare, indicating that clinician failure to administer the measures is the most likely cause of missed assessments. This is consistent with the discrepancy in completion rates, which is likely due to the intervals at which assessments are completed in each setting. MBC assessments are expected for every intake appointment and each continuation/maintenance group, making the collection of those data more routine. Weekly MBC assessments are gathered every Monday of IOP, so missing data are unlikely unless a patient fails to attend a scheduled Monday IOP session but does attend IOP later in the week (and in this case, patients are directed to complete MBC measures when they return to IOP). For weekly outpatient treatment, however, MBC data are only gathered monthly; thus, clinicians must track when clients completed their last MBC assessment to determine whether each session requires MBC administration.

## Lessons learned and future directions

The use of MBC with suicidal youth has numerous benefits. Based on our findings, clinicians find the use of MBC in this setting acceptable and beneficial, and results suggest that MBC can be implemented with reasonable fidelity even in primarily clinical (rather than research) settings. Our findings regarding staff perceptions of MBC were consistent with prior research identifying key reasons to use (or not use) progress monitoring in psychotherapy [66], as well as qualitative research with clinician stakeholders [67–69].

The STAR-Center team has implemented several strategies, in addition to those outlined above, to improve the utility and acceptability of MBC. Critically, at least one (and presently two) STAR-Center clinical staff are designated to provide additional feedback and support of the MBC system as a part of their professional duties. These staff coordinate twice monthly meetings to bridge communication between clinical staff, researchers, analysts, and programmers affiliated with STAR-Center, which facilitate identification of clinical needs not yet served by MBC and problem solving around obstacles to MBC implementation. Clinical staff liaisons play an essential role in enhancing clinician commitment to MBC, serving as advocates for the clinical staff and promoting an environment of enthusiasm and positivity about MBC. We have found that having designated leadership among clinical staff in support of the MBC program is critical to the long-term success of MBC at STAR-Center, consistent with recommendations from extant literature regarding organizational supports for MBC [70].

**Fig 2:**
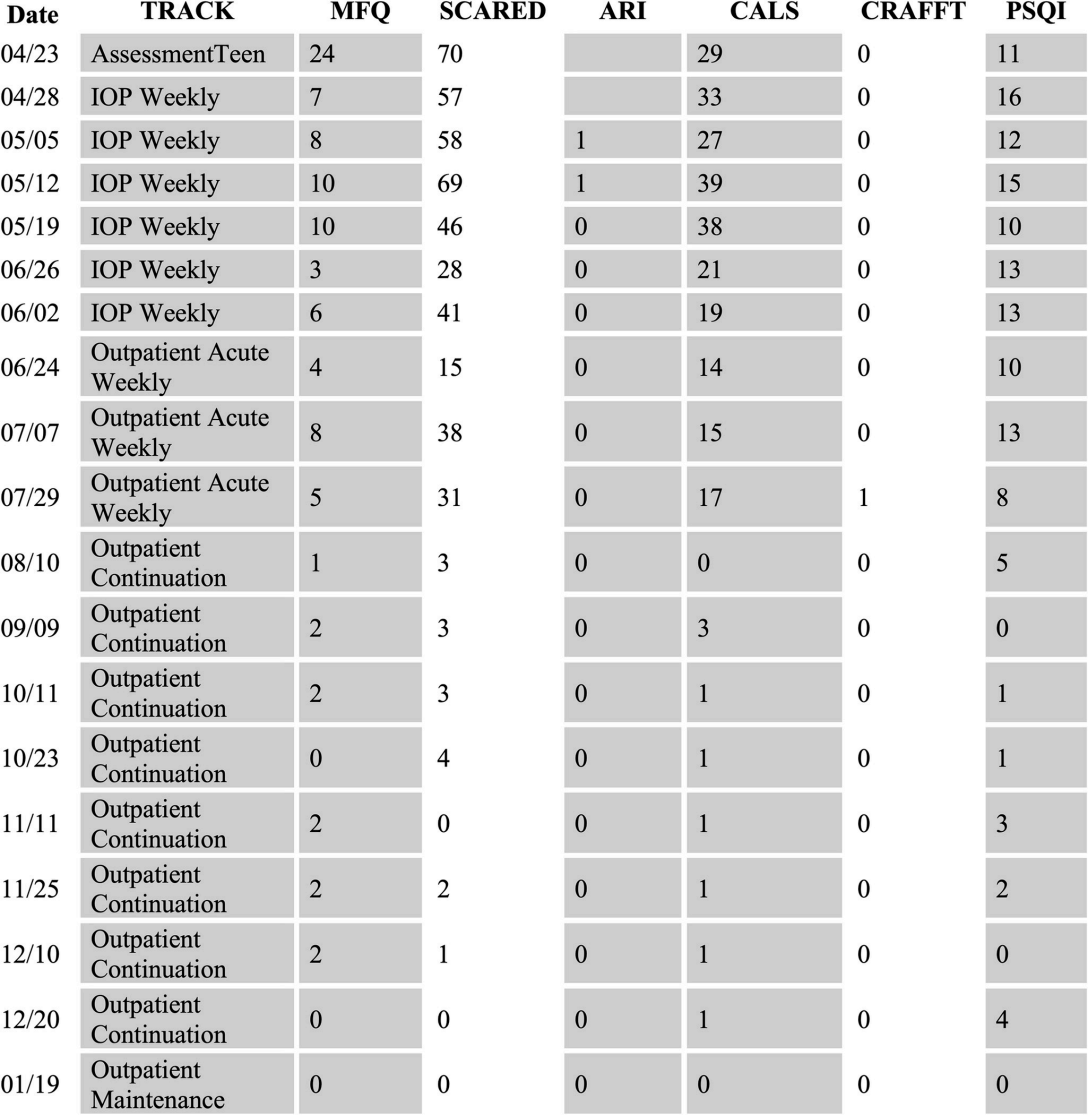
De-identified example of MBC data display on the clinician dashboard. This is an example of a display for a single patient followed over time. MFQ = Short Mood and Feelings Questionnaire; SCARED = Screen for Child Anxiety Related Disorders; CALS = Children’s Affective Lability Scale; ARI = Affective Reactivity Index; CRAFFT = Car, Relax, Alone, Forget Friends, Trouble; PSQI = Pittsburgh Sleep Quality Index.

As evidenced by sparser MBC completion in the context of weekly outpatient care described above, ensuring clinicians gather MBC data at irregular intervals can be difficult. At STAR-Center, MBC assessments are not integrated into the EMR system, which makes routine reminders more complex; these data are instead tracked through a clinician dashboard in an external system (see Fig 2). Although the independent nature of the STAR-Center’s MBC system has some benefits, such as the ability to highly tailor its workflow, measures, and output to meet the clinic’s needs, it also has some drawbacks, such as a separate interface, the need for clinicians to initiate MBC assessments (e.g., generate and then text or email a link to youth/caregiver), and the inability to populate the EMR directly with MBC-generated data (although screenshots can be copied into clinical notes as appropriate). A future direction for STAR-Center leadership involves the design and implementation of an acceptable reminder system for clinicians to request patient-completed MBC assessments in weekly outpatient treatment and to complete clinician-rated MBC assessments at necessary intervals.

Although STAR-Center leadership is committed to the use of MBC with its patients, MBC systems are not without costs, both financial and with respect to staff time and effort. MBC systems that are more complex (e.g., allow for different measures to be administered to different patients, “flagging” high-risk responses, integration with EMR data) require significant resources, which hinders MBC implementation in many clinical environments. Policy changes that provide financial support for these programs, such as increased reimbursement rates from third-party payers for services that involve MBC, could increase the likelihood of widespread MBC adoption in mental healthcare settings. Finally, greater research is needed to examine not only the feasibility of MBC in routine clinical care for suicidal youth, but also to elucidate the ways that MBC impacts patient outcomes.
